# Loss of RhoA Exacerbates, Rather Than Dampens, Oncogenic K-Ras Induced Lung Adenoma Formation in Mice

**DOI:** 10.1371/journal.pone.0127923

**Published:** 2015-06-01

**Authors:** Inuk Zandvakili, Ashley Kuenzi Davis, Guodong Hu, Yi Zheng

**Affiliations:** 1 Experimental Hematology and Cancer Biology, Cincinnati Children’s Hospital Medical Center, Cincinnati, Ohio, United States of America; 2 Molecular and Developmental Biology Graduate Program, Cincinnati Children’s Hospital Medical Center, Cincinnati, Ohio, United States of America; 3 Medical-Scientist Training Program, College of Medicine, The University of Cincinnati, Cincinnati, Ohio, United States of America; Children's Hospital Boston, UNITED STATES

## Abstract

Numerous cellular studies have indicated that RhoA signaling is required for oncogenic Ras-induced transformation, suggesting that RhoA is a useful target in Ras induced neoplasia. However, to date very limited data exist to genetically attribute RhoA function to Ras-mediated tumorigenesis in mammalian models. In order to assess whether RhoA is required for K-Ras-induced lung cancer initiation, we utilized the K-Ras^G12D^ Lox-Stop-Lox murine lung cancer model in combination with a conditional RhoA^flox/flox^ and RhoC^-/-^ knockout mouse models. Deletion of the floxed *Rhoa* gene and expression of K-Ras^G12D^ was achieved by either CCSP-Cre or adenoviral Cre, resulting in simultaneous expression of K-Ras^G12D^ and deletion of RhoA from the murine lung. We found that deletion of RhoA, RhoC or both did not adversely affect normal lung development. Moreover, we found that deletion of either RhoA or RhoC alone did not suppress K-Ras^G12D^ induced lung adenoma initiation. Rather, deletion of RhoA alone exacerbated lung adenoma formation, whereas dual deletion of RhoA and RhoC together significantly reduced K-Ras^G12D^ induced adenoma formation. Deletion of RhoA appears to induce a compensatory mechanism that exacerbates adenoma formation. The compensatory mechanism is at least partly mediated by RhoC. This study suggests that targeting of RhoA alone may allow for compensation and a paradoxical exacerbation of neoplasia, while simultaneous targeting of both RhoA and RhoC is likely to lead to more favorable outcomes.

## Introduction

In the United States, lung cancer kills more people each year than breast, prostate and colon cancer combined [1]. Lung adenocarcinoma is the most common subtype of lung cancer and often harbors activating mutations of K-Ras [2]. K-Ras is a founding member of the Ras GTPase superfamily and is a key signal transduction protein that integrates extracellular stimuli and promotes cell proliferation and survival. Activating mutations of K-Ras disrupt the GTPase activity of the protein, increasing levels of GTP-bound K-Ras, which results in continuous signaling. Despite being among the very first oncogenes discovered, direct pharmacological inhibition of K-Ras has remained elusive [3]. An alternative strategy to inhibiting K-Ras directly is to target its downstream signaling pathways. While the RAF-MEK-ERK signaling pathway is the most important transducer of K-Ras signaling, several other downstream signaling axes including the PI3K-AKT-mTOR pathway, Ral GTPases, and the Rho family of GTPases have each been implicated as required elements for Ras induced transformation.

The mammalian Rho GTPase family includes over 20 members, of which RhoA, Rac1 and Cdc42 are among the best characterized. These Rho GTPases regulate the cell cycle and actin cytoskeleton and are thus critical regulators of processes such as cell shape, adhesion, migration, polarity and proliferation [4]. Given these essential functions of Rho GTPases and the availability of pre-clinical and clinical inhibitors of Rho GTPase signaling, they pose an important topic in cancer research [5–8]. Importantly, Rho GTPases have been shown to be critical for Ras-induced transformation of fibroblasts and epithelial cells [9–13]. Over a decade ago, several classic studies in the GTPase field demonstrated that blocking RhoA signaling could suppress Ras-induced transformation, and conversely that constitutively active RhoA could cooperate synergistically with Raf to promote cell transformation [9,12,13]. Additionally, studies have shown that RhoA plays an important permissive role in cell cycle progression through the G_1_-S phase: namely, increased RhoA activity inhibits p21^Waf1/Cip1^ and results in increased amounts of cyclin D1 and p27^Kip1^ [14–16]. Indeed, blockage of RhoA signaling is thought to induce INK4 activity, in turn halting the cell cycle [16]. Thus, the plurality of evidence shows RhoA is a positive regulator of the cell cycle. Subsequently, RhoA has been found to be either overexpressed or hyperactive in a variety of cancers, and RhoA activity is correlated with negative outcomes in gastric, hepatocellular, esophageal squamous cell, breast and lung carcinomas [17–22].

More recently, greater attention has been paid to the role of Rac1 in tumorigenesis due to the availability of murine genetic models. These studies have confirmed a positive role of Rac1 in tumor initiation. Rac1 was found to be required for K-Ras-induced lung adenoma formation in mice and a Rac1 splicing variant with increased activity, Rac1b, appears to promote tumorigenesis in this context [23,24]. Two other recent studies have indicated that pharmacological inhibition of the downstream mediators of RhoA signaling, ROCK and FAK, are promising therapeutic targets [25,26]. However, to date no mammalian genetic studies have directly assessed whether RhoA is required for oncogenic Ras-mediated tumorigenesis.

In the present work, we have sought to investigate the role of RhoA and the closely related Rho GTPase, RhoC, in K-Ras-induced tumorigenesis using a well-established inducible K-Ras^G12D^ knock-in mouse model of lung adenoma induction in combination with our RhoA conditional knockout, RhoA^flox/flox^, and RhoC^-/-^ mouse models. Our investigation of these genetic studies produced surprising results that individually RhoA and RhoC are dispensable for the oncogenic K-Ras induced lung tumorigenesis and loss of RhoA alone exacerbates, rather than suppresses, tumor initiating activity. Our study implies that pharmacological targeting of multiple Rho family members, e.g. RhoA and RhoC in this context, is required to prevent tumorigenesis.

## Results

We first sought to determine whether mice would be viable without RhoA expression in their bronchiolar epithelium. We crossed a RhoA conditional mouse (RhoA^flox/flox^) with mice harboring the Club Cell Secretory Promoter Cre (CCSP-Cre), as outlined in Table 1. This promoter is expressed mainly in Club cells, eponymously referred to as Clara cells, which are a major component of the bronchial epithelium [27].

**Table 1:** Breeding Schematic for Transgenic Mice.

	Transgenes			
Shorthand	Rho^WT^	RhoA^cKO^	RhoC^-/-^	Double Knockout (DKO)
RhoA genotype	RhoA^+/+^	RhoA^flox/flox^	RhoA^+/+^	RhoA^flox/flox^
RhoC genotype	RhoC^+/+^	RhoC^+/+^	RhoC^Δ2–3^	RhoC^Δ2–3^
**Additional Transgenes:**				
K-Ras knock-in	LSL-K-Ras^G12D^			
Cre-recombinase	Adeno-Cre or CCSP-Cre			

**Fig 1:**
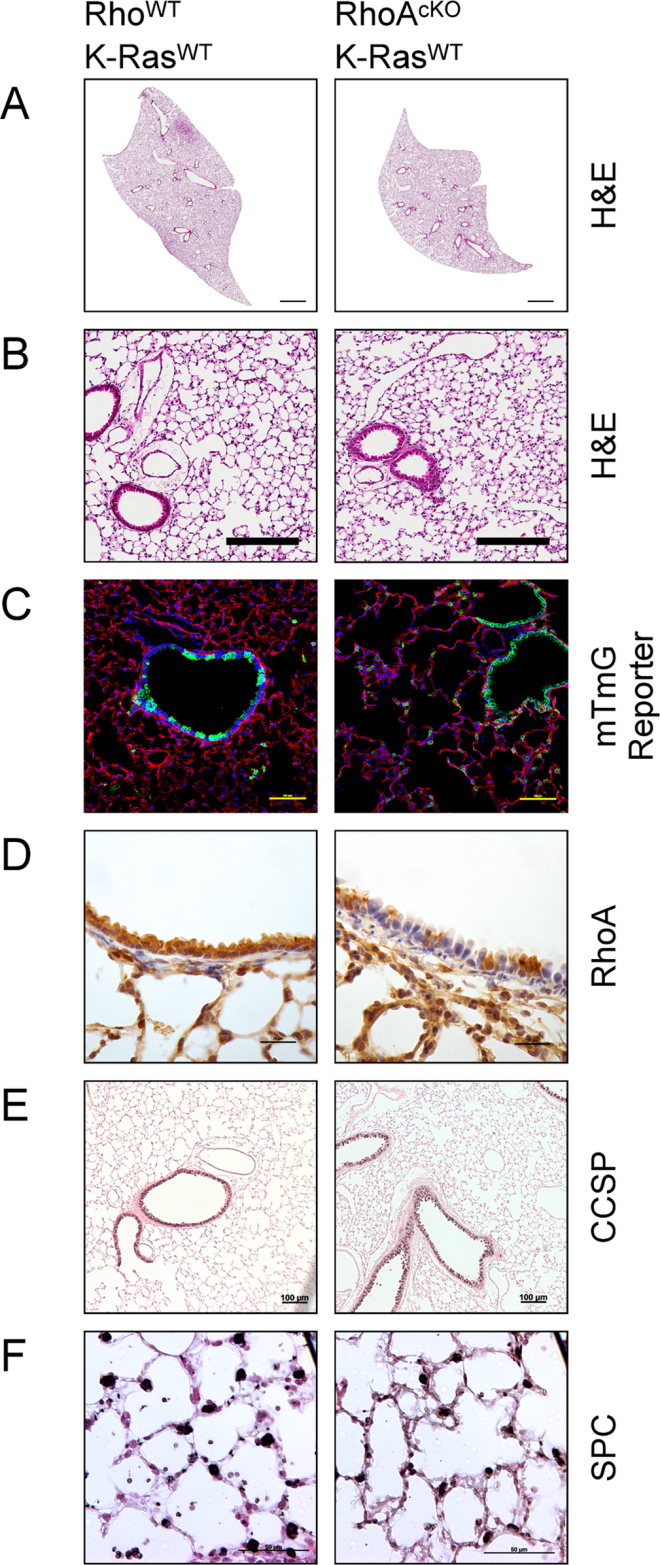
CCSP-promoter driven deletion of RhoA does not affect normal lung development. Rho^WT^ and RhoA^flox/flox^ mice were mated with CCSP-Cre mice on a K-Ras^WT^ background. **(A & B)** Lungs by H&E (bars represent 1mm and 200μm for panels A & B respectively). **(C)** Mice were crossed to a tdTomato to eGFP reporter line to assess for difference is Cre-expression pattern (bars represent 100μm). Green represents eGFP and Cre activity. Red presents tdTomato and the absence of Cre activity. Blue represents DAPI staining. **(D)** Immunohistochemistry for RhoA (brown) with hematoxylin counterstain (bars represent 25μm). **(E)** Immunohistochemistry for CCSP (black), counterstained with nuclear fast red (bars represent 100μm). **(F)** Immunohistochemistry for SPC (black), counterstained with nuclear fast red (bars represent 50μm).

Mice harboring CCSP-Cre RhoA^flox/flox^ transgenes were born at the expected Mendelian ratio, did not differ in weight during weaning or adulthood and lived as long as Cre-negative littermates. These mice did not display any signs of overt respiratory distress. Their lungs were normal appearing both grossly and microscopically (Fig 1A and 1B). We wondered whether the Cre expression would be altered by RhoA deletion so we crossed these mice to the double-reporter “mTmG” mouse. These mice constitutively express tdTomato, which switches to eGFP with the expression of Cre-recombinase [28]. We found Cre expression in a patchy distribution within bronchiolar epithelium, which was similar between RhoA^cKO^ and Rho^WT^ backgrounds (Fig 1C).

We next assessed for the penetrance of RhoA deletion. Using immunohistochemistry, we found RhoA was deleted from the bronchioles in a patchy pattern similar to the distribution of eGFP in the reporter mice (Fig 1D). Recombination of the RhoA^flox/flox^ allele was also evident in RhoA^cKO^ via PCR with RhoA deletion specific primers (S1 Fig). Moreover, we did not find any qualitative differences in the expression pattern of Club cells or Type II alveolar cells as assessed by CCSP and SPC staining, respectively (Fig 1E and 1F). Thus, deletion of RhoA from Club cells by the CCSP-Cre resulted in viable mice with no signs of respiratory dysfunction. There were no overt differences between RhoA^cKO^ mice and control mice with regards to lung architecture or the amount and distribution of major lung cell types.

**Fig 2:**
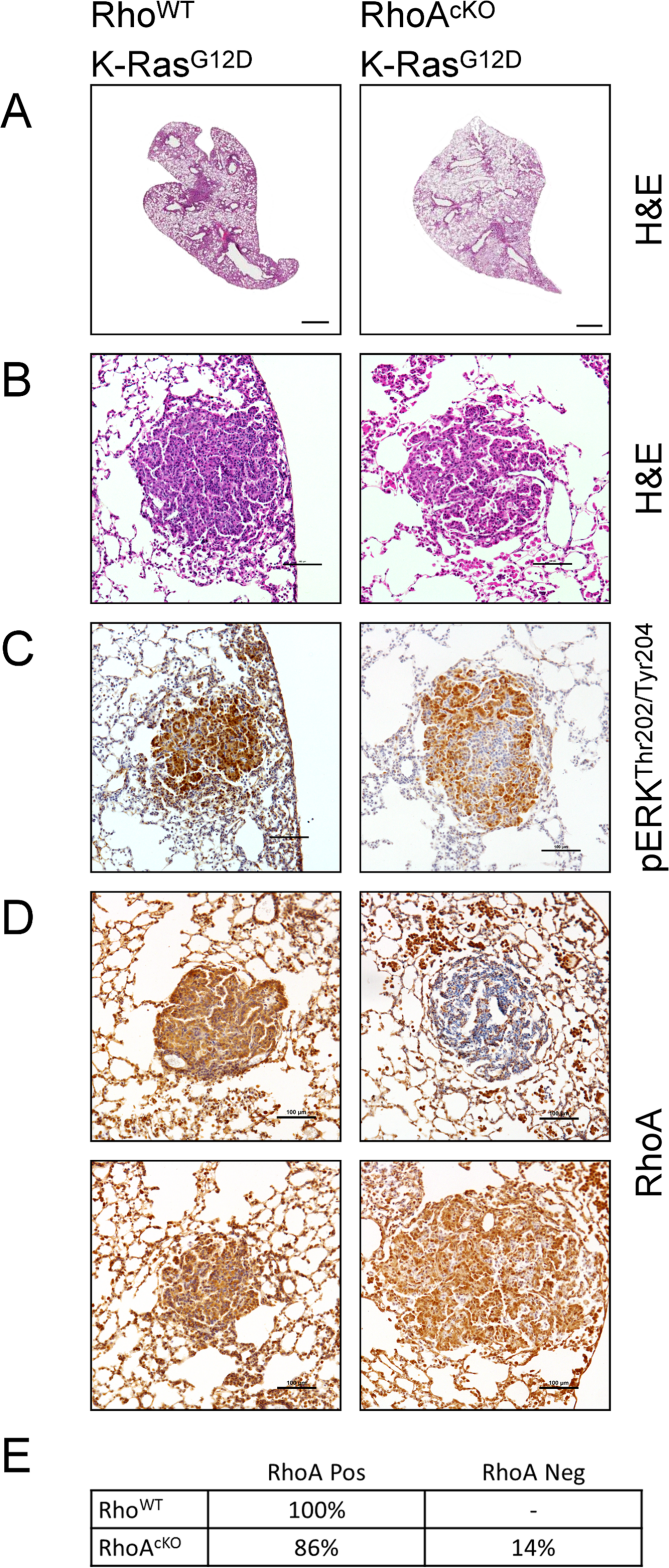
RhoA is not essential for CCSP-promoter driven, K-Ras^G12D^-induced, lung adenoma formation in mice. Rho^WT^ and RhoA^flox/flox^ mice were mated with CCSP-Cre mice on a K-Ras^G12D^ background. **(A & B)** K-Ras^G12D^ lungs by H&E (bars represent 1mm and 200μm for panels A & B respectively). **(C)** Immunohistochemistry for pERK^Thr202/Tyr204^ (brown) with hematoxylin counterstain (bars represent 100μm). **(D)** Immunohistochemistry for RhoA (brown) with hematoxylin counterstain (bars represent 100μm). **(E)** Quantification of the RhoA-status of adenomas as assessed by immunohistochemistry. Greater than 30 tumors were counted from four mice per group.

Next we sought to determine whether RhoA is required for oncogenic K-Ras-induced tumor initiation *in vivo*. We bred the Lox-STOP-Lox K-Ras^G12D^ (LSL-K-Ras^G12D^) transgene into the CCSP-Cre;RhoA^flox/flox^ mice. At least two different constructs of CCSP-Cre (also referred to as CC10-Cre) have been successfully used to induce lung tumors in mice [29–32]. It was also previously shown that CCSP-Cre LSL-K-Ras^G12D^ mice develop a strong inflammatory component to their disease with abundant infiltration of alveolar macrophages and neutrophils [29,30]. Consistent with previous works, we found the CCSP-Cre;LSL-K-Ras^G12D^;RhoA^flox/flox^ mice to have grossly abnormal lungs with abundant infiltration and adenoma formation (Fig 2A and 2B). These mice also displayed prominent hyperplasia of the bronchiolar epithelium and atypical adenomatous hyperplasia (AAH) [33]. We did not find any differences in the disease pathology of RhoA^cKO^ mice in comparison to Rho^WT^ mice and both groups formed adenomas. Only a proportion of the adenomas in either group stained positively for pERK^Thr202/Tyr204^, in agreement with previous reports (Fig 2C) [26,34]. Immunohistochemical staining of the lungs of these mice demonstrated positive RhoA staining for all adenomas in the Rho^WT^ group, whereas a fraction of adenomas stained positively in the RhoA^cKO^ group (Fig 2D and 2E). Although only ~15% of the adenomas observed were RhoA-null in the RhoA^cKO^ group, we found numerous RhoA-null hyperplastic growths, including AAH (S2 Fig).

Given that most adenomas evaded RhoA-deletion by CCSP-Cre, our results imply that there is selection pressure for the maintenance of RhoA signaling during KRas^G12D^-induced transformation. Our results are similar to the finding that Rac1 is required for KRas-induced adenoma formation by Kissil et al. [23]. In their findings, Kissil et al. utilized a similar approach with Rac1^flox/flox^ transgenic mice and found the Rac1 locus was never recombined, and always remained undeleted in adenomas. Our findings differ in that we do find RhoA-null adenomas, thus demonstrating that RhoA signaling is not essential for oncogenic K-Ras-induced adenoma formation.

**Fig 3:**
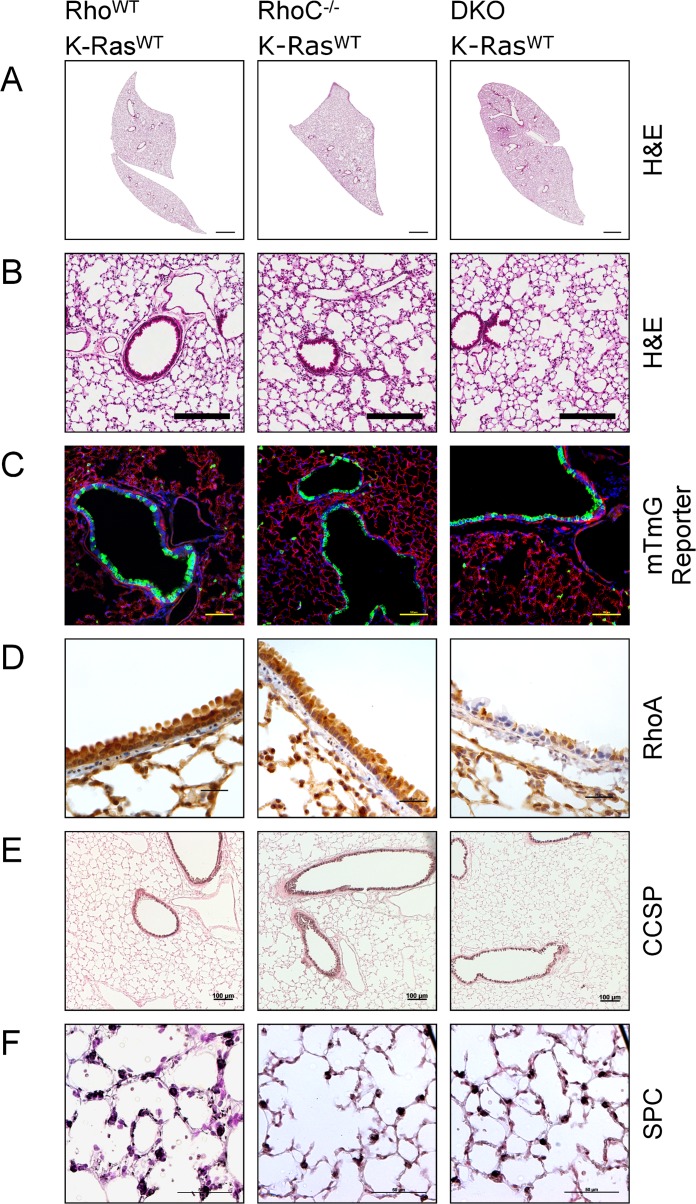
Deletion of RhoA and RhoC together does not impair normal lung development. Rho^WT^, RhoC^-/-^ and DKO mice were mated with CCSP-Cre mice on a K-Ras^WT^ background. **(A & B)** Lungs by H&E (bars represent 1mm and 200μm for panels A & B respectively). **(C)** Mice were crossed to a tdTomato to eGFP reporter line to assess for difference is Cre-expression pattern (bars represent 100μm). Green represents eGFP and Cre activity. Red presents tdTomato and the absence of Cre activity. Blue represents DAPI staining. **(D)** Immunohistochemistry for RhoA (brown) with hematoxylin counterstain (bars represent 25μm). **(E)** Immunohistochemistry for CCSP (black), counterstained with nuclear fast red (bars represent 100μm). **(F)** Immunohistochemistry for SPC (black), counterstained with nuclear fast red (bars represent 50μm).

We wondered if other closely related Rho GTPases may be redundant for RhoA function, and decided to investigate RhoC as a candidate since RhoC shares a very high degree of sequence homology with RhoA and is also thought to be pro-tumorigenic [35]. We did not investigate the third Rho subfamily member, RhoB, because gene targeting and other studies have shown it plays an opposite function to RhoA [36–39]. As previously reported, constitutively RhoC null mice, RhoC^-/-^ mice, are viable and do not demonstrate any overt phenotype [40]. Similar to RhoA deletion alone, CCSP-Cre;RhoA^flox/flox^;RhoC^-/-^ double-knockout (DKO) mice were born at the expected Mendelian ratio, did not display any signs of overt respiratory distress and their lungs were grossly normal (Fig 3A and 3B). When crossed with the mTmG reporter line we found no differences in Cre expression between Rho^WT^, RhoC^-/-^ or DKO mice (Fig 3C). We found RhoA expression, as assessed by immunohistochemistry, to be similar between RhoC^-/-^ and Rho^WT^ mice (Fig 3D). RhoA expression in double-knockout mice was similar to that of RhoA^cKO^ mice (Fig 3D). Specifically, we found RhoA deletion from the bronchiolar epithelium in DKO mice to be in a patchy distribution (Fig 3D). Effective RhoA-locus recombination was also evident by PCR (S1 Fig). We did not find any qualitative differences in the expression pattern of Club cells or Type II alveolar cells in any of these mice as assessed by CCSP and SPC staining, respectively (Fig 3E and 3F). Thus, dual RhoA and RhoC deletion from a large proportion of the bronchiolar epithelium does not have any substantial effect on normal lung development or function.

**Fig 4:**
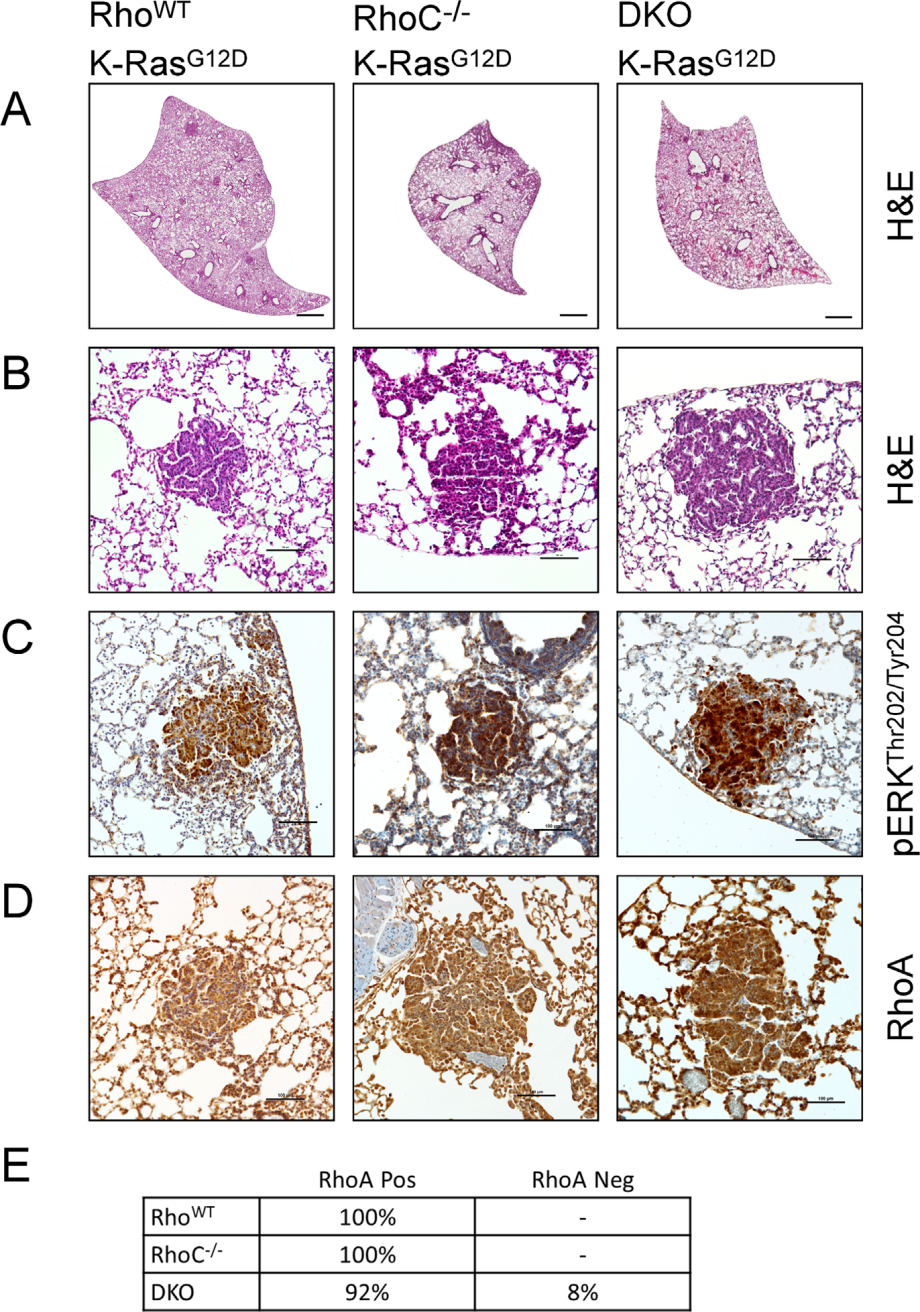
Neither RhoA nor RhoC is required for K-Ras^G12D^-induced adenoma formation in a CCSP-Cre model. Rho^WT^, RhoC^-/-^ and DKO mice were mated with CCSP-Cre mice on a K-Ras^G12D^ background. **(A & B)** K-Ras^G12D^ lungs by H&E (bars represent 1mm and 200μm for panels A & B respectively). **(C)** Immunohistochemistry for pERK^Thr202/Tyr204^ (brown) with hematoxylin counterstain (bars represent 100μm). **(D)** Immunohistochemistry for RhoA (brown) with hematoxylin counterstain (bars represent 100μm). **(E)** Quantification of the RhoA-status of adenomas as assessed by immunohistochemistry. Greater than 30 tumors were counted from four mice per group.

We further assessed whether double deletion of RhoA and RhoC would affect K-Ras^G12D^ induced adenoma formation. We crossed RhoA^flox/flox^;RhoC^-/-^ mice with the CCSP-Cre;LSL-K-Ras^G12D^ line and observed adenoma formation in both RhoC^-/-^ and DKO mice (Fig 4A and 4B). It was previously shown that RhoC^-/-^ mouse embryonic fibroblasts are able to form Ras-induced colonies in agar, and that RhoC^-/-^ mice can form MMTV-PyMT induced breast tumors [40], but to the best of our knowledge this is the first time RhoC has been shown to be dispensable for Ras-induced tumor formation *in vivo*. We found that these adenomas stained positively for pERK^Thr202/Tyr204^ by immunohistochemistry (Fig 4C). Additionally, immunohistochemistry for RhoA showed that almost all adenomas in the RhoC^-/-^ and DKO stained positively for RhoA (Fig 4D and 4E). These results raise the possibility that there is a strong selective disadvantage for RhoA deletion during adenoma formation in the absence of RhoC. While a RhoA deletion band is present by PCR (S1 Fig), it may be derived from heterozygous RhoA lox recombination or non-adenoma tissue. Deletion of either RhoA or RhoC did not result in differences in Ki67 or cleaved-caspase 3 staining of adenomas suggesting that RhoA and RhoC status do not affect cell proliferation or survival (S2 Fig)

Importantly, we found that “RhoA-null” tumors in DKO mice maintained pMLC^Ser20^ staining, suggesting compensatory activity for RhoA loss (S3 Fig). Taken together, these results indicate that while RhoA and RhoC are each dispensable for K-Ras^G12D^ mediated tumorigenesis, together they contribute to adenoma formation.

**Fig 5:**
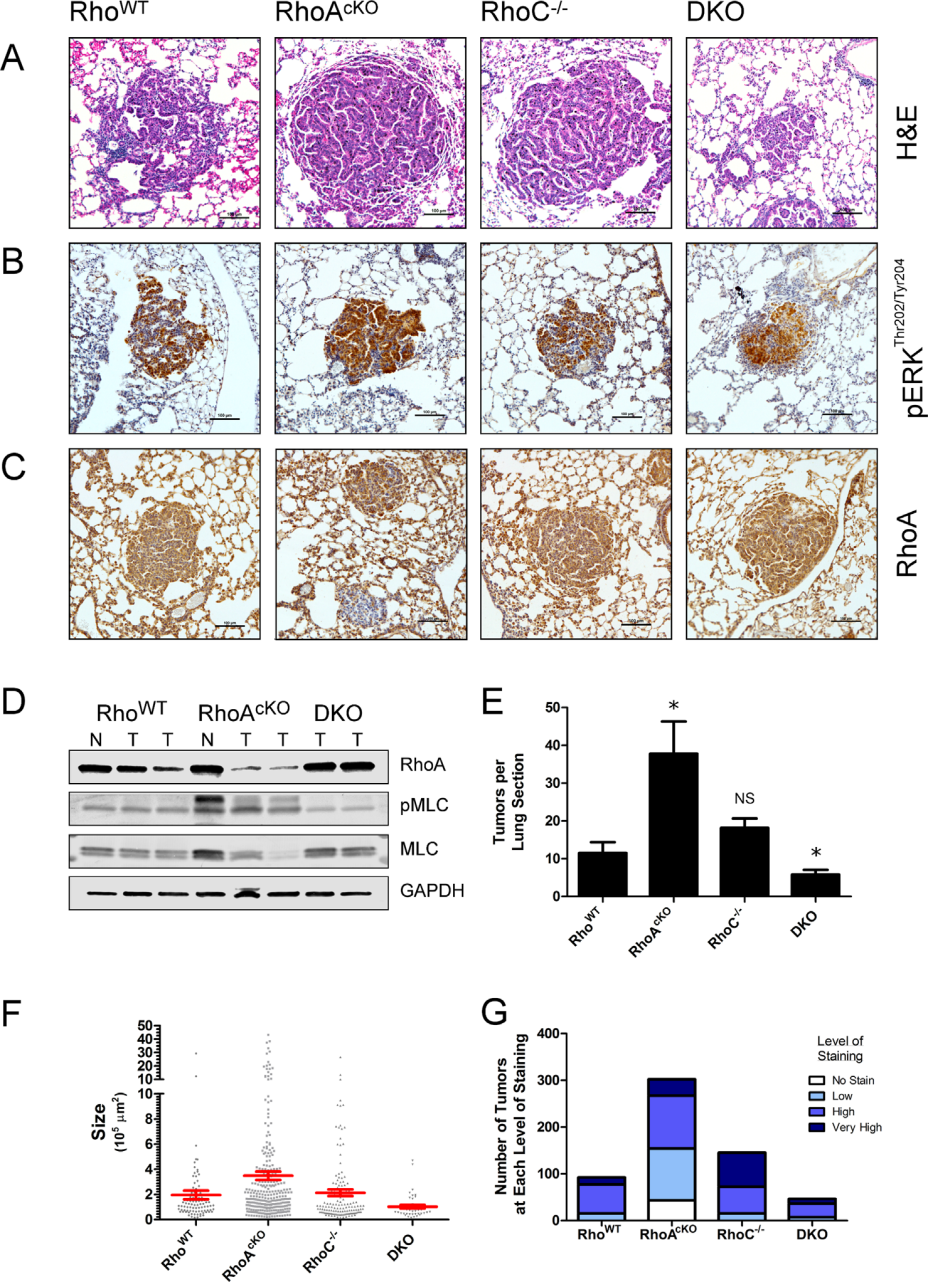
RhoA and RhoC are important in combination, but not individually, for K-Ras^G12D^ induced sporadic lung adenoma formation. Mice from different Rho backgrounds were administered Adeno-Cre virus endotracheally. Lungs were harvested after 12 weeks. **(A)** H&E of adenomas (bars represent 100μm). **(B)** Immunohistochemistry for pERK^Thr202/Tyr204^ (brown) with hematoxylin counterstain (bars represent 100μm). **(C)** Immunohistochemistry for RhoA (brown) with hematoxylin counterstain (bars represent 100μm). **(D)** Western blots of microdissected tumors (N = normal lung, T = tumor). **(E)** Quantification of tumor burden. Means represent the quantification of four separate, evenly spaced and similarly sized lung sections, from four mice for each group (n = 4, * = p ≤ 0.001). Data representative of two separate experiments. **(F)** Quantification of tumor sizes. Data points represent the area of individual tumors expressed in μm^2^. Red bars represent the mean tumor area. **(G)** Proportion of RhoA-positive and RhoA-null tumors. Displayed as RhoA staining by the level of staining intensity (levels are as follows: no staining; normal = same intensity staining as adjacent normal alveoli; high = greater than adjacent normal alveoli; very high = much greater intensity staining than adjacent normal alveoli).

To eliminate potential developmental effects of K-Ras^G12D^ and RhoA deletion during mouse development, as well as to achieve more robust RhoA deletion, we next used adenoviral mediated induction of lung adenomas to yield a sporadic tumor model [41,42]. Six- to eight-week-old mice were administered intratracheal Cre-expressing adenovirus (Adeno-Cre) in order to simultaneously induce oncogenic K-Ras and delete the floxed *Rhoa* gene. Twelve weeks post-adenoviral induction mice were euthanized and their lungs harvested. Hematoxylin and eosin (H&E) staining demonstrated adenoma formation in Rho^WT^, RhoA^cKO^, RhoC^-/-^ and DKO mice (Fig 5A). As before, only a proportion of the adenomas stained positively for pERK^Thr202/Tyr204^ in each group (Fig 5B). Immunohistochemical staining revealed positive RhoA staining in all adenomas from the Rho^WT^, RhoC^-/-^ and DKO groups (Fig 5C), whereas only a subset of adenomas from the RhoA^cKO^ group expressed RhoA (Fig 5C and 5D). Therefore, similar to the CCSP-Cre model, RhoA-null adenomas were able to form by Adeno-Cre induction in K-Ras^G12D^;RhoA^flox/flox^ mice, indicating RhoA alone is not essential for K-Ras^G12D^ mediated tumorigenesis.

We quantified the tumor burden of the Adeno-Cre mice via serial sectioning of their lungs. Surprisingly, we found that there were many more adenomas in the RhoA^cKO^ group as compared to the control, Rho^WT^ group (Fig 5E), and in addition to quantity, many adenomas in the RhoA^cKO^ group are larger in size than those in controls (Fig 5F). Interestingly, analysis of the RhoA status of the larger adenomas within the RhoA^cKO^ group revealed that the relatively larger adenomas (>10,000 μm^2^) were ones in which RhoA was not fully deleted. Quantification of the number and size of tumors revealed similar numbers and sizes of tumors between Rho^WT^ and RhoC^-/-^ mice (Fig 5E and 5F). However, DKO mice had fewer adenomas and these tumors were smaller than those formed in either Rho^WT^ or RhoC^-/-^ mice (Fig 5E and 5F). Interestingly, we found noticeably increased amounts of hyperplastic lesions in the RhoA^cKO^ group (S4 Fig). Similar to the CCSP-Cre model, increased hyperplasia did not translate to differences in Ki67 or cleaved-caspase 3 staining of adenomas regardless of RhoA status (S4B and S4C Fig).

Due to the heterogeneity of RhoA-positive and RhoA-null adenomas in the RhoA^cKO^ mouse group, we subdivided tumors based on their RhoA deletion status in our analysis. The RhoA expression status of each adenoma was quantified by immunohistochemistry. We found that all adenomas in the Rho^WT^, RhoC^-/-^ and DKO groups expressed RhoA in the Adeno-Cre induction model (Fig 5G). In particular, every adenoma found in the DKO group retained RhoA and escaped recombination of the RhoA^flox/flox^ allele (Fig 5G) whereas a portion of the tumors in the RhoA^cKO^ group was completely depleted of RhoA, similar to that of the CCSP-Cre model (Fig 5D and 5G). However, the sum of the RhoA-null tumors in the RhoAcKO group did not account for the overall increased numbers of tumors in this group (Fig 5G). This data suggests that there is a strong selection advantage to RhoA expression in the absence of RhoC during K-Ras^G12D^-driven adenoma formation.

Finally, we microdissected Adeno-Cre induced adenomas from mice with different Rho backgrounds and assessed downstream signaling via Western blot (Fig 5D). Both RhoA and RhoC are known to bind to and signal via ROCK and regulate phosphorylation of MLC [43,44]. We found that RhoA-null adenomas maintained or upregulated pMLC^Ser19^ signaling, suggesting likely compensation by RhoC or other related Rho GTPases. The upregulated levels of pMLC^Ser19^ in RhoA^cKO^ mice correlate with the increased tumorigenesis in these mice. Moreover, we found that pMLC^Ser19^ levels are not increased in DKO mice, correlating with the observation that these mice have both smaller and fewer tumors than Rho^WT^, RhoA^cKO^ or RhoC^-/-^ background mice. These observations are consistent with the interpretation that there is a selection pressure to maintain downstream pMLC signaling when RhoA signaling is lost.

## Discussion

Several previous studies of the relationship between RhoA and Ras signaling in cell transformation prompted our current investigation of whether RhoA or RhoC are required for K-Ras-induced tumor formation [9–11]. The main readouts of the earlier studies were anchorage-independent growth in soft agar, cell proliferation, decreased requirement of serum for growth, and focus formation in the Petri dish. These studies addressed two important questions: whether increased RhoA signaling could lead to transformation of cells and whether abrogation of RhoA signaling could prevent Ras-induced transformation. To answer the first question, several studies reported that over expression of wild type RhoA, or constitutively active RhoA or Rac1, can weakly transform cells, suggesting an oncogenic role of active RhoA [9,10,12,13,45,46]. Transformation of cells expressing active Raf were synergistically enhanced with constitutively active RhoA [9]. Answering the second question, it was found that dominant negative forms of RhoA can block Ras-induced transformation [9,10]. However, these classical cell biology studies were limited by the nature of the approaches—dominant-negative or constitutively active mutant over expression—by the models—mostly fibroblasts and tumor cell lines—and by the *in vitro* nature of the readouts. Nevertheless, these works laid out the conventional rationale that RhoA is a proto-oncogene product and RhoA targeting may be of therapeutic value [35].

Our investigation focuses on addressing the role of RhoA in K-Ras-driven tumorigenesis *in vivo* in established mouse models of lung cancer. First, it appears that neither RhoA nor RhoC is required for normal lung function in mice. Second, our data shows K-Ras^G12D^-induced adenomas can form in the absence of RhoA, indicating that RhoA is not required for K-Ras^G12D^-induced tumorigenesis. Third, the closely related RhoA homolog, RhoC, which has been implicated as an important pro-tumor metastasis molecule, is also dispensable for K-Ras^G12D^-induced tumorigenesis. Fourth, we found an increase in the numbers of adenomas in the RhoA^cKO^ sporadic lung tumor model in which RhoA is deleted by adenoviral expression of Cre. Fifth, an attempt at combined deletion of RhoA and RhoC resulted in reduced adenoma formation and retention of RhoA in all adenomas. These results indicate that there is a selective advantage to retain RhoA in the absence of RhoC, and there is likely a redundant and/or compensatory role of RhoC in RhoA signaling downstream of oncogenic K-Ras in adenoma formation. Consistent with this interpretation, the phosphorylation status of MLC, downstream of RhoA and RhoC signaling, was not decreased in either RhoA or RhoC-null adenomas, nor in adenomas from DKO mice, in which RhoA is retained.

Our counterintuitive findings are in line with several recent studies that have begun to challenge the conventional paradigm that RhoA is a simple proto-oncogene. Two whole-exome sequencing studies of human lymphomas found recurrent Gly17Val mutations in RhoA which confer a dominant negative or loss of function phenotype [47,48]. Another very recent study using two different mouse models of colon cancer found that mice carrying a dominant negative RhoA transgene (RhoA^T19N^) had increased tumor burden [49]. Furthermore, a study of K-Ras-driven liver tumorigenesis in Zebrafish has found that an active form of RhoA (RhoA^G14V^) hindered tumor formation whereas dominant-negative RhoA (RhoA^T19N^) accelerated tumorigenesis [50]. While the pro-oncogenic mechanism of RhoA function loss remains unclear, our work suggests that other Rho family members such as RhoC may compensate for RhoA loss, or possibly even “overcompensate”, resulting in a paradoxical increase in tumorigenesis.

A limitation of our mouse model in this study is that RhoA is deleted at the same time as K-Ras^G12D^ is expressed, and thus our work does not address whether RhoA plays a role in tumor maintenance or progression. It is possible that once oncogenic K-Ras induced tumors have developed, they become addicted to permissive signaling from wild-type RhoA. In this case, RhoA signaling would be required for tumor maintenance. Indeed, an examination of human cancer cell lines treated with RhoA-specific siRNA or the Rho inhibitor Rhosin shows that they are dependent on RhoA function for proliferation and progression (data not shown). These findings pose an intriguing possibility that targeted inhibition of RhoA activity alone is useful for suppressing advanced tumors in certain instances, but may exacerbate preneoplastic lesions. Another caution interpreting our results is that both mouse cancer models are accompanied by significant inflammatory responses which could potentially be modulated by RhoA status, which in turn may be promoting the inflammatory component (i.e. effects through tumor microenvironment).

The interplay between RhoA and RhoC signaling is in some ways similar to that of B-Raf and c-Raf in oncogenic K-Ras-driven adenoma initiation. Recent studies have found that c-Raf, but not B-Raf, is required for K-Ras-driven adenoma formation, despite historical evidence in mouse embryonic tissues and fibroblasts suggesting the opposite, that B-Raf but not c-Raf is required for ERK phosphorylation [51–55]. In fact, similar to our findings, pharmacologic targeting of B-Raf results in increased activity of c-Raf and a paradoxical increase in ERK phosphorylation and tumorigenesis in KRas-mutant cancers [53,56–58]; though this is not the case with genetic deletion of B-Raf. Interestingly, Blasco *et al*. also found that whole organism deletion of both B-Raf and c-Raf resulted in no obvious phenotype in mice, also similar to our finding of overtly healthy mice with dual deletion of RhoA and RhoC from the bronchiolar epithelium [51].

Our study of the relationship between RhoA, RhoC and K-Ras in murine lung adenoma formation sheds light on the broader subject of cellular signal transduction networks, and how signal redundancy and compensation in these networks can result in counterintuitive results when the network is modified. These insights are important considering the increased interest and emphasis on targeted therapeutics against individual pathways. The robustness of signal transduction networks paired with the evolutionary power of clonal selection in cancer suggests the best antineoplastic strategies may need to incorporate multiple drug targets with an anticipation of how cancer cells will adapt to and evade targeted therapies. Our study suggests that simultaneous targeting of both RhoA and RhoC signaling is critical, as targeting of RhoA alone may worsen clinical outcomes. Further study confirming our proposed compensatory mechanism is warranted, as is a deeper understanding of the overlapping and differential functions of RhoA, RhoC and other related Rho GTPases in cancer biology. Last, our study suggests that targeting ROCK signaling, which is a shared downstream mediator of both RhoA and RhoC, may be sufficient to block K-Ras-driven cancer as has been previously suggested [25,59].

## Materials and Methods

### Animal work

RhoA^flox/flox^ mice generated as previously described [60]. RhoC^Δ2–3^, LSL-K-Ras, CCSP-Cre and mTmG mice have been previously characterized [28,29,40,61]. Mice were bred and housed in a specific pathogen-free vivarium at Cincinnati Children’s Hospital Medical Center and protocols were approved by the Institutional Animal Use and Care Committee of the Cincinnati Children’s Hospital Research Foundation (IACUC2013-0069), in compliance with the Association for Assessment and Accreditation of Laboratory Animal Care and the Office of Laboratory Animal Welfare. All *in vivo* experiments were performed using age-matched mice. Genotyping primers can be found in the supplementary information (S1 Table). Adeno-Cre virus (Ad5-CMV-Cre) was obtained from the University of Iowa Gene Transfer Vector Core. Administration of Adeno-Cre virus was performed as previously described using a dose of 10^8^ PFU per mouse [41]. Mice were anesthetized with isoflurane and pain control was achieved with buprenorphine.

### Western blotting

Western blotting was performed using standard protocols, with important changes or steps described herein. Briefly, lysis was performed using radioimmunoprecipitation assay buffer (RIPA buffer) supplemented with protease inhibitors cocktail (cOmplete Protease Inhibitor Cocktail Tablets, Roche) and phosphatase inhibitor cocktail consisting of sodium fluoride, sodium orthovanadate, sodium pyrophosphate, ß-glycerophosphate and sodium molybdate (Simple Stop 1 Phosphatase Inhibitor Cocktail, Gold Biotechnology). Transfer was performed using the Trans-Blot Turbo Transfer System (Bio-Rad). Visualization was performed using fluorescently conjugated secondary antibodies and an infrared imaging System (LiCor Odyssey CLx).

### Immunohistochemistry

Briefly, tissues were fixed using 3.7% paraformaldehyde in PBS overnight and embedding to paraffin. Secondary antibodies and horse-radish peroxidase (HRP) used consisted of either Signal Stain Boost (Cell Signaling Technology) or anti-rabbit donkey secondary (GE Healthcare) and Vectastain Elite ABC Kit (Vector Labs) avidin-biotin complex. The developing reagent was DAB (3, 3’-diaminobenzidine) HRP substrate (Vector Labs). Counterstaining was performed using Gill’s hematoxylin or nuclear fast red. Quantification of stainings were made by serially sectioning lungs in 250μm step increments and counting a sample of evenly spaced sections for the number of tumors. Tumor size was expressed as tumor area as measured in ImageJ.

### Primary antibodies

Primary antibodies for immunoblotting or immunohistochemistry are as follows: RhoA (67B9) Rabbit mAb (1:400 dilution, Cell Signaling Technology [CST] #2117), Myosin Light Chain 2 (D18E2) Rabbit mAb (CST #8505), Phospho-Myosin Light Chain 2 (Ser19) Mouse mAb (1:150 dilution, CST #3675), Phospho-p44/42 MAPK (Erk1/2) (Thr202/Tyr204) (D13.14.4E) Rabbit mAb (CST #4370), Phospho-MEK1/2 (Ser221) (166F8) Rabbit mAb (CST #2338), GAPDH (D16H11) Rabbit mAb (CST #5174), Anti-Myosin Light Chain (phospho S20) antibody (Abcam ab2480), Ki67 Rabbit mAb, (790–4286 Ventana), cleaved-caspase 3 Rabbit Ab (orb137850 Biorbyt), N-Terminal Pro SPC Rabbit Ab (Seven Hills Bioreagents WRAB-9337), and CCSP Rabbit Ab (Seven Hills Bioreagents WRAB-3950). All antibodies were used at the manufacturers recommended dilutions unless otherwise stated.

### Data analysis and presentation

Data was gathered and analyzed in either ImageJ 1.47 (NIH), GraphPad Prism (GraphPad) or the GNU Image Manipulation Program 2.8 (GIMP). Analysis in GraphPad Prism comprised of unpaired two-way student T-tests or ANOVA.

## Supporting Information

S1 Fig PCR analysis of mouse genotypes and lox deletion bands. DNA was isolated from mouse lungs of different Rho background mice mated to either CCSP-Cre K-Ras^WT^ (K-WT) or CCSP-Cre LSL-K-Ras^G12D^ mice (KRas). Pairs of K-Ras^WT^ and LSL-K-Ras^G12D^ mice for each Rho background are shown. Deletion bands for LSL-K-Ras^G12D^ mice demonstrate expression of K-Ras^G12D^ (deletion band is the larger band at 315bp). Deletion bands for RhoA demonstrate recombination of the RhoA^flox/flox^ locus (deletion band at 667bp). Yellow arrows point to the 500bp band of a 100bp ladder.
(TIFF)

S2 Fig CCSP-promoter driven hyperplastic growths and adenomas. **(A)** RhoA immunohistochemistry (brown) of hyperplastic lesions with hematoxylin counterstain of CCSP-Cre LSL-K-Ras^G12D^ mice from different Rho backgrounds (bars represents 100μm). **(B)** Percentage of Ki67 positive cells in CCSP-Cre LSL-K-Ras^G12D^ driven adenomas (n = 4, p >0.05). **(C)** Representative image of cleaved-caspase 3 (CC3) staining of CCSP-Cre LSL-K-Ras^G12D^ driven adenomas. Positive control is injured renal tubules (20X). Bars represent 100μm.
(TIFF)

S3 Fig Downstream K-Ras and RhoA signaling status in CCSP-promotor driven adenomas. Immunohistochemistry of serially sectioned adenomas from CCSP-Cre LSL-K-Ras^G12D^ mice. Rows represent serial sections of the same adenoma from their respective Rho backgrounds. Columns are of RhoA, pMLC^Ser20^, pMEK^Ser221^ and pERK^Thr202/Tyr204^ stainings respectively with hematoxylin counterstain (bars represents 100μm).
(TIFF)

S4 Fig Adeno-Cre induced hyperplastic growths and adenomas. **(A)** RhoA immunohistochemistry (brown) with hematoxylin counterstain of Adeno-Cre induced LSL-K-Ras^G12D^ mice from different Rho backgrounds (bars represents 100μm). **(B)** Percentage of Ki67 positive cells in Adeno-Cre LSL-K-Ras^G12D^ driven adenomas (n = 4, p >0.05). **(C)** Representative image of cleaved-caspase 3 (CC3) staining of Adeno-Cre LSL-K-Ras^G12D^ driven adenomas. Positive control is injured renal tubules (20X). Bars represent 100μm.
(TIFF)

S5 Fig Original Western Blots. (TIFF)

S1 Table Genotyping and deletion primers. Table of genotyping and deletion primers used for PCR. Specific PCR protocols can be found in the references for transgenic mice under Materials and Methods.
(DOCX)
