# Supplementary figures and images for: Loss of RhoA Exacerbates, Rather Than Dampens, Oncogenic K-Ras Induced Lung Adenoma Formation in Mice
Source: PLoS One. 2015 Jun 1;10(6):e0127923. doi: 10.1371/journal.pone.0127923 (PMC4452309; doi:10.1371/journal.pone.0127923)

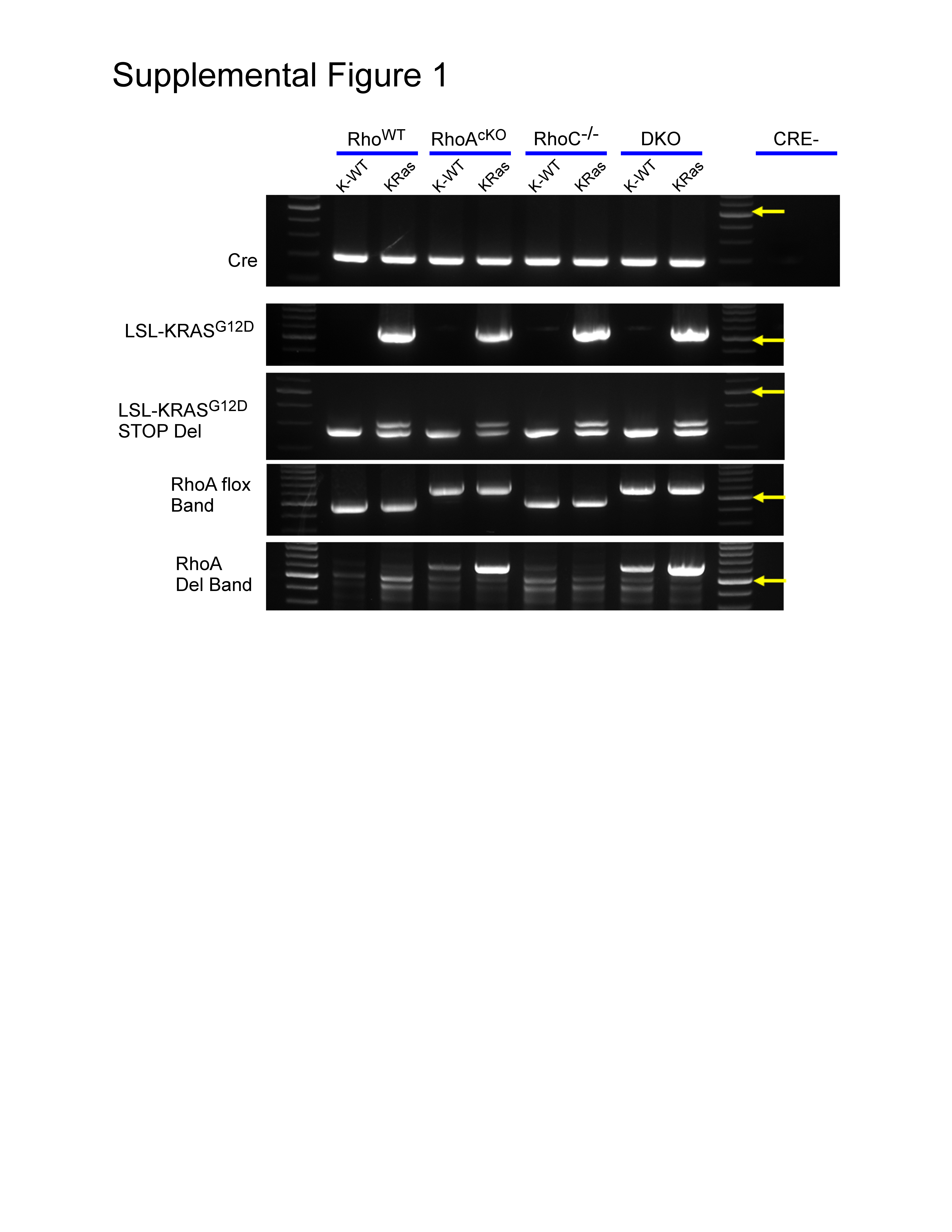

Supplement: S1 Fig — DNA was isolated from mouse lungs of different Rho background mice mated to either CCSP-Cre K-RasWT (K-WT) or CCSP-Cre LSL-K-RasG12D mice (KRas). Pairs of K-RasWT and LSL-K-RasG12D mice for each Rho background are shown. Deletion bands for LSL-K-RasG12D mice demonstrate expression of K-RasG12D (deletion band is the larger band at 315bp). Deletion bands for RhoA demonstrate recombination of the RhoAflox/flox locus (deletion band at 667bp). Yellow arrows point to the 500bp band of a 100bp ladder. (TIFF) [file pone.0127923.s001.tiff]

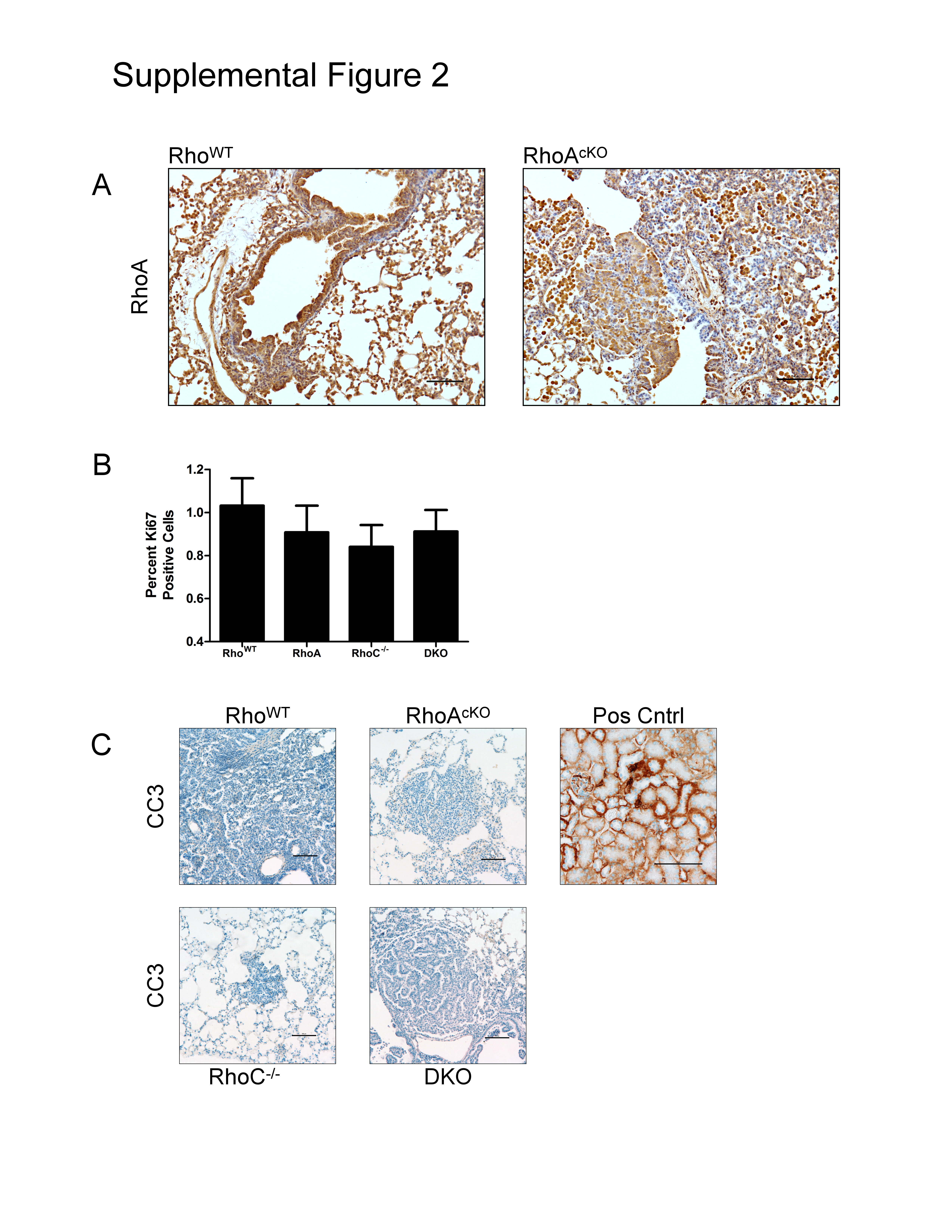

Supplement: S2 Fig — (A) RhoA immunohistochemistry (brown) of hyperplastic lesions with hematoxylin counterstain of CCSP-Cre LSL-K-RasG12D mice from different Rho backgrounds (bars represents 100μm). (B) Percentage of Ki67 positive cells in CCSP-Cre LSL-K-RasG12D driven adenomas (n = 4, p >0.05). (C) Representative image of cleaved-caspase 3 (CC3) staining of CCSP-Cre LSL-K-RasG12D driven adenomas. Positive control is injured renal tubules (20X). Bars represent 100μm. (TIFF) [file pone.0127923.s002.tiff]

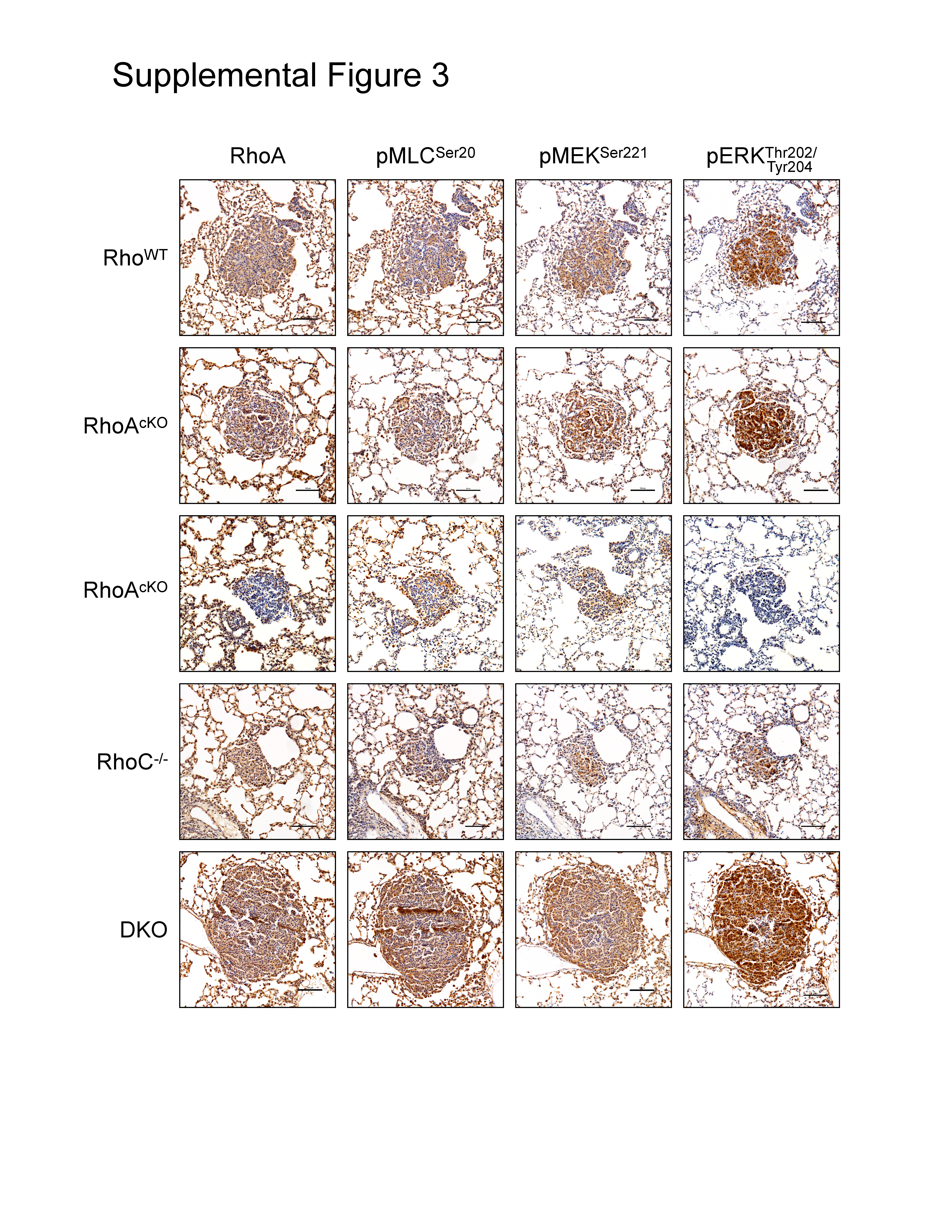

Supplement: S3 Fig — Immunohistochemistry of serially sectioned adenomas from CCSP-Cre LSL-K-RasG12D mice. Rows represent serial sections of the same adenoma from their respective Rho backgrounds. Columns are of RhoA, pMLCSer20, pMEKSer221 and pERKThr202/Tyr204 stainings respectively with hematoxylin counterstain (bars represents 100μm). (TIFF) [file pone.0127923.s003.tiff]

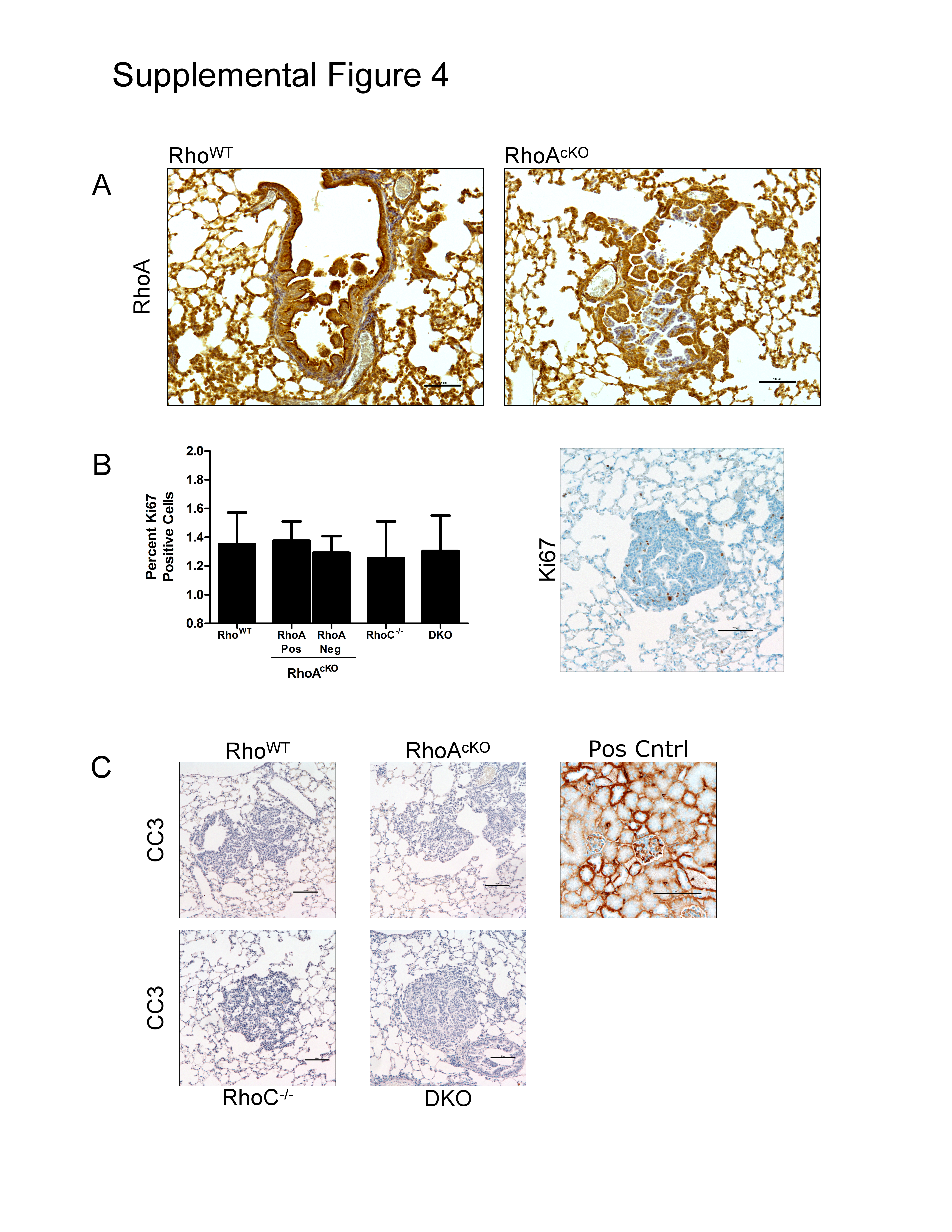

Supplement: S4 Fig — (A) RhoA immunohistochemistry (brown) with hematoxylin counterstain of Adeno-Cre induced LSL-K-RasG12D mice from different Rho backgrounds (bars represents 100μm). (B) Percentage of Ki67 positive cells in Adeno-Cre LSL-K-RasG12D driven adenomas (n = 4, p >0.05). (C) Representative image of cleaved-caspase 3 (CC3) staining of Adeno-Cre LSL-K-RasG12D driven adenomas. Positive control is injured renal tubules (20X). Bars represent 100μm. (TIFF) [file pone.0127923.s004.tiff]

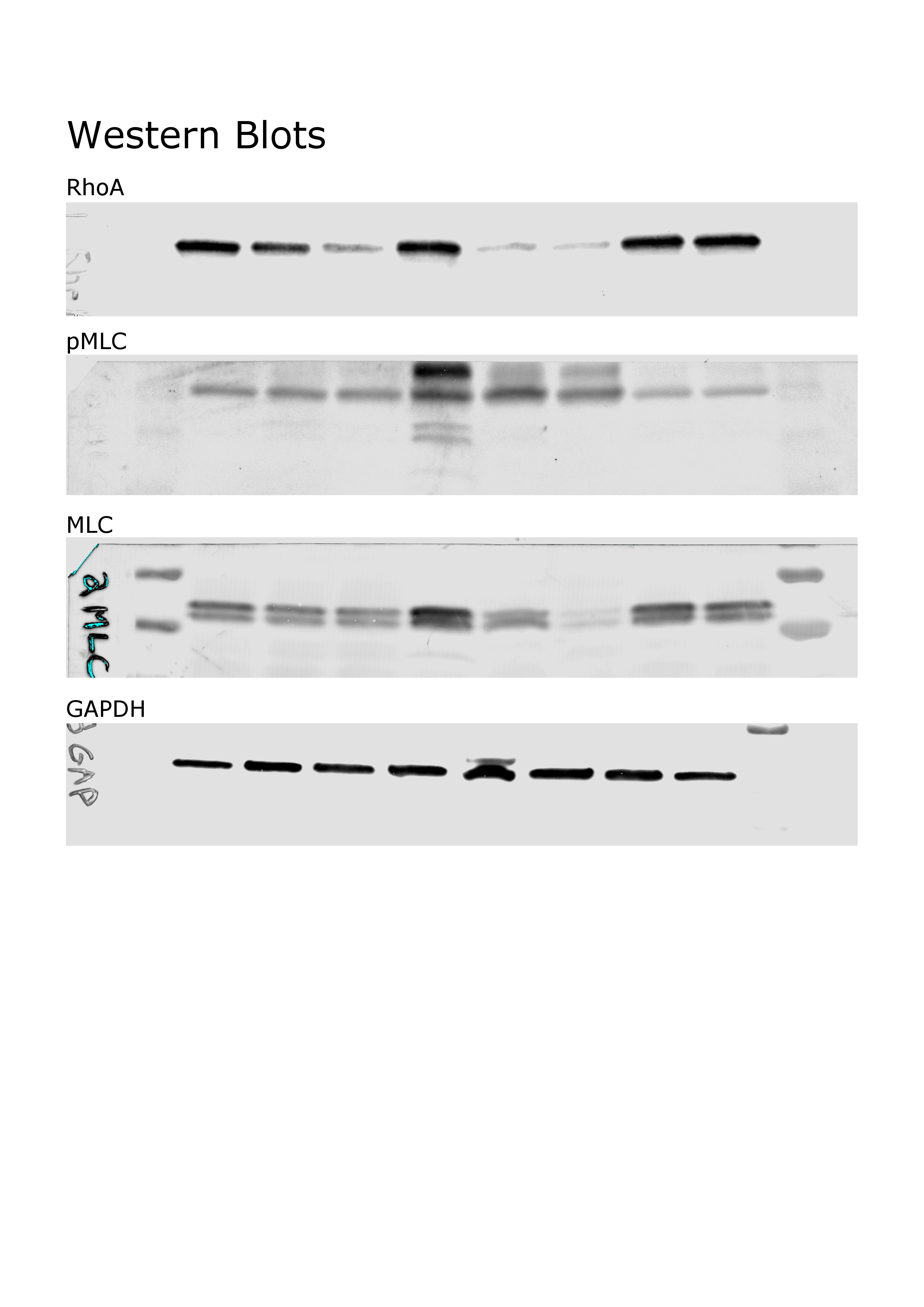

Supplement: S5 Fig — (TIFF) [file pone.0127923.s005.tiff]
