# Supplementary material for: Loss of RhoA Exacerbates, Rather Than Dampens, Oncogenic K-Ras Induced Lung Adenoma Formation in Mice
Source: PLoS One. 2015 Jun 1;10(6):e0127923. doi: 10.1371/journal.pone.0127923 (PMC4452309; doi:10.1371/journal.pone.0127923)
Supplement: S1 Table — Table of genotyping and deletion primers used for PCR. Specific PCR protocols can be found in the references for transgenic mice under Materials and Methods. (DOCX) [file pone.0127923.s006.docx]

**Table S1: Genotyping and deletion primers**

| Universal Cre | F 5’-GAT TTC GAC CAG GTT CGT TC-3’  R 5’-GCT AAC CAG CGT TTT CGT TC-3’ |
| --- | --- |
| LSL-K-Ras^G12D^ Genotyping | F 5’-CGC AGA CTG TAG AGC AGC G-3’  R 5’-CCA TGG CTT GAG TAA GTC TGC-3’ |
| LSL-K-Ras^G12D^ Deletion | F 5'-GGG TAG GTG TTG GGA TAG CTG-3'  R 5'-TCC GAA TTC AGT GAC TAC AGA TGT ACA GAG-3’ |
| RhoA^flox/flox^ Genotyping | F 5’-TCT CTG CAC TGA GGG AGT TAG G-3’  R 5’-GTA CAT ACA GGG AAT GGA AAC AAG G-3’ |
| RhoA^flox/flox^ Deletion | F 5'-GCA CTG AGG GAG TTA GG-3’  R 5'-CTA CAC TAG CTG GGC AC-3’ |
| RhoC^-/-^ | Univ. 5’-TCC AGG ACC CCA TGC AAA GGT-3’  WT 5’-CAC AGG CAC CGT CAC CCA CAA-3’  MUT 5’-AGG TCC CTC GAC CTG CAG CC-3’ |
